# The Ever‐Expanding Influence of the Endothelial Nitric Oxide Synthase

**DOI:** 10.1111/bcpt.70029

**Published:** 2025-03-27

**Authors:** Riham Rafea, Mauro Siragusa, Ingrid Fleming

**Affiliations:** ^1^ Institute for Vascular Signalling, Centre for Molecular Medicine Goethe University Frankfurt am Main Germany; ^2^ Partner Site RheinMain German Center for Cardiovascular Research (DZHK) Frankfurt am Main Germany

**Keywords:** eNOS, eNOS phosphorylation, metabolism, RNA editing, *S*‐nitrosylation

## Abstract

Nitric oxide (NO) generated by the endothelial NO synthase (eNOS) plays an essential role in the maintenance of vascular homeostasis and the prevention of vascular inflammation. There are a myriad of mechanisms that regulate the activity of the enzyme that may prove to represent interesting therapeutic opportunities. In this regard, the kinases that phosphorylate the enzyme and regulate its activity in situations linked to vascular disease seem to be particularly promising. Although the actions of NO were initially linked mainly to the activation of the guanylyl cyclase and the generation of cyclic GMP in vascular smooth muscle cells and platelets, it is now clear that NO elicits the majority of its actions via its ability to modify redox‐activated cysteine residues in a process referred to as *S*‐nitrosylation. The more wide spread use of mass spectrometry to detect *S*‐nitrosylated proteins has helped to identify just how large the NO sphere of influence is and just how many cellular processes are affected. It may be an old target, but the sheer impact of eNOS on vascular health really justifies a revaluation of therapeutic options to maintain and protect its activity in situations associated with a high risk of developing cardiovascular disease.

**Summary**

- Nitric oxide is generated by the endothelial cells that form the innermost lining of blood vessels.
- This molecule helps maintain vascular health by targeting and chemically modifying numerous proteins in a process referred to as *S*‐nitrosylation.
- Impaired functioning of the endothelial nitric oxide synthase that generates nitric oxide has been directly linked to a heightened risk of developing cardiovascular disease.
- New methods to detect the chemical modification have helped expand the list of proteins targeted by nitric oxide, as well as the cellular processes affected.

## 1 Introduction

The role of endothelium‐derived nitric oxide (NO) in vascular homeostasis is well documented as it is important for preventing endothelial cell as well as platelet activation and maintaining vascular smooth muscle cell quiescence. There have been numerous reports demonstrating that defective activation of the endothelial NO synthase (eNOS) and reduced NO production, particularly in combination with increased free radical production, have a deleterious effect on vascular homeostasis and promote endothelial cell activation and adhesion molecule expression [1, 2]. On the other hand, a genetic predisposition to enhanced NO generation and soluble guanylyl cyclase (sGC) activation is associated with a reduced risk of coronary heart disease, peripheral artery disease and stroke [3]. The best‐studied pathways linking NO with vascular disease have focussed on sGC and nuclear factor‐κB (NF‐κB) activation but the impact of NO on the cardiovascular system are not limited to these two mechanisms alone. Indeed, NO can react with cysteine residues to modulate protein function, a posttranslational modification referred to as *S‐*nitrosation (or *S‐*nitrosylation). Numerous proteins were initially found to be *S‐*nitrosylated in the presence of endogenous or exogenous NO sources but better detection methods have helped to document that *S‐*nitrosylation is a highly regulated posttranslational modification in native cells. Indeed, the detailed characterization of the impact of *S‐*nitrosylation on protein function has demonstrated that it affects a large number of cellular processes, from cell metabolism and gene expression to cell viability [4, 5]. In this work, we discuss the regulation of eNOS expression and activity, describe the mechanisms controlling *S‐*nitrosylation and highlight its impact on protein function.

## 2 eNOS Activity

NOS enzymes synthesize NO and L‐citrulline from L‐arginine, NADPH and O_2_. Essential cofactors in this process are calmodulin (CaM), flavin mononucleotide (FMN) and tetrahydrobiopterin (BH_4_). The active eNOS enzyme is a homodimer that requires a Zn(4S) cluster for dimerization [6]. Each monomer is composed of a C‐terminal reductase domain that contains flavin and is structurally similar to P450 reductases and an N‐terminal oxygenase domain that contains the catalytic haem group. The two regions in each monomer are linked by a CaM‐binding region, and it is the presence of this region that differentiates the Ca^2+^‐dependent NOS isoforms (eNOS and neuronal NOS) from the Ca^2+^‐independent inducible NOS [7]. In order to generate NO, electrons must be moved from NADPH through the reductase domain via FAD and FMN to the oxygenase domain haem iron (in the opposite monomer [8]) where O_2_ binds and activates it [9]. The shuttling of electrons through the reductase domain requires a physical movement or pivoting of the entire FMN‐binding site in eNOS [10]. Importantly, the movement of this insert is also regulated by the binding of Ca^2+^/CaM [10, 11], which induces a conformational change that controls the transfer of electrons from the reductase domain to haem [12, 13, 14, 15]. A detailed study on nNOS also proposed that the physical movement of the FMN‐binding domain could be altered by serine and tyrosine phosphorylation [16]. Shortly thereafter, a tyrosine residue in the FMN‐binding domain of eNOS (Tyr657) was reported to regulate NO production, and when it was phosphorylated, it abrogated enzyme activity by preventing FMN binding [17, 18, 19].

The oxygenase domain of eNOS contains the active site of the enzyme where NO generation takes place and has binding sites for haem, BH_4_ and L‐arginine. BH_4_ has several different functions; it stabilizes the eNOS dimer [20], shields the Zn(4S) from oxidants [6] and acts as an allosteric modulator of arginine binding. It also converts the haem iron from a low‐spin to a high‐spin state (for detailed review, see [21]). eNOS uncoupling is the process that occurs in the absence of BH_4_, and the lack of the final electron donor means that the transport of electrons to ferrous‐haem–O_2_ species generated during the stepwise activation of O_2_ by NOS does not occur fast enough to prevent their oxidative decay [22, 23]. Other modifications may contribute to the process as BH_4_ deficiency alone is reportedly sufficient to reduce intracellular GSH:GSSG ratio and cause eNOS *S*‐glutathionylation [24]. The process of eNOS uncoupling results in the generation of O_2_^−^ and subsequently peroxynitrite (ONOO^−^) to initiate the oxidative stress that contributes to endothelial cell inflammatory activation that precedes atherogenesis [22, 23]. For a while, the eNOS‐catalysed generation of O_2_^−^ was attributed to the monomerization of the enzyme [25]. However, the NADPH oxidase activity is limited and the uncoupling of eNOS is not likely to be associated with reversible monomerization and it is now apparent that eNOS dimer formation in vivo is essentially insensitive to BH_4_ levels [26, 27].

Several interacting proteins are required for optimal eNOS function, in addition to CaM and caveolin 1, which is important for regulation of eNOS at the plasma membrane [28, 29, 30]. The molecular chaperone heat shock protein 90 (Hsp90) is particularly important as it can promote eNOS activation [31], in addition to its role in the folding of the enzyme and the insertion of haem into the immature protein [32]. The large influence of Hsp90 can be attributed to its role as a major signalling hub. Indeed, Hsp90 binds many of the kinases, for example, Akt and Pyk2 known to directly regulate eNOS phosphorylation and activity. Several additional proteins have been identified in the eNOS interactome under basal conditions and altered in conditions associated with endothelial dysfunction (for reviews, see [1, 2, 33]).

## 3 Posttranslational Modifications

The eNOS protein is posttranslationally modified by a number of mechanisms. The C terminus is both *N*‐myristoylated and *S‐*palmitoylated, which plays a role in its interactions with the Golgi membrane and cholesterol‐rich microdomains of the plasma membranes [34, 35, 36]. It can also be acetylated, glycosylated, glutathionylated, phosphorylated and *S‐*nitrosylated [37]. The reversible *S‐*glutathionylation of eNOS is implicated in its uncoupling to generate O_2_^−^ rather than NO to contribute to oxidative stress–induced changes in vascular reactivity [38, 39]. In endothelial cells, there are reports of eNOS being modified by *S‐*nitrosylation (on Cys92 and Cys97 in the zinc‐tetrathiolate cluster at the eNOS homodimer interface), a modification that was reported to determine the stability of the active dimer and be inversely related to the phosphorylation of Ser1177 [40, 41, 42]. eNOS nitrosylation reversibly attenuates enzyme activity [43] and has been proposed to account (at least partly) for the actions of oxidized LDL on NO generation. It is unlikely that the NO required for this reaction is derived from the normally functioning eNOS enzyme; rather, the high levels of NO generated by the inducible NOS (iNOS) seem to account for this phenomenon.

Dynamic changes in the phosphorylation of eNOS have proven to be crucial for its activation. For example, the enzyme is basally phosphorylated on Ser and Thr residues [44], but there is a rapid reshuffling of phosphorylated sites that accompanies enzyme activation. Ca^2+^‐elevating agonists are particularly effective in inducing the dephosphorylation of Thr495 in the CaM‐binding domain of eNOS, a step that is essential for CaM to bind to the enzyme [45, 46]. The best‐studied eNOS phosphorylation site is Ser1177 and is targeted by a number of kinases including Akt, the AMP‐activated protein kinase, protein kinase A and CaM kinase II (for review, see [47]). Although the phosphorylation of the latter site is often used as a surrogate index of eNOS activation, this is an error as although phosphorylation does increase electron flow through the reductase domain to increase NO production [48, 49, 50], it is not essential for enzyme activation. This was particularly well demonstrated in a study in which phosphomimetic and nonphosphorylatable Ser1176 (murine sequence) eNOS mutant mice were generated [51]. Although exchanging serine with aspartate did increase basal and stimulated endothelial NO production and replacement with alanine did have a slight hypertensive effect, the consequences of interfering with Ser1176 phosphorylation on agonist‐induced NO generation and vascular relaxation were small [51]. There are also reports of agonists, for example, insulin, that effectively increase the phosphorylation of Ser1177 without increasing NO production [17, 52]. Several additional serine phosphorylation sites have been identified in eNOS including Ser633 (also located in the FMN‐binding domain), which seems to be particularly sensitive to shear stress [53], as well as Ser114 and Ser615—the functions of which have yet to be fully elucidated [2, 47].

That eNOS is phosphorylated on tyrosine residues has been known for quite a while [29, 44] and was initially linked to its interaction with caveolin 1 [29], but the exact phosphorylation site(s) were elusive. Indeed, the modification was less robust than that of serine and threonine, probably because of the loss of critical tyrosine kinases in endothelial cells in culture [17]. Most is known about the consequences of phosphorylating Tyr657 (human sequence) in the FMN‐binding domain, as this abrogates enzymatic activity by preventing the binding of FMN to inhibit electron flow through the reductase domain [17, 19]. One kinase implicated in this modification is proline‐rich tyrosine kinase (Pyk2), a redox‐regulated kinase activated in vascular cells undergoing oxidative stress as well as by insulin. Indeed, it is the phosphorylation of eNOS on Tyr657 by Pyk2 that can account for the inability of insulin to increase NO production in native endothelial cells despite the concomitant activation of Akt and phosphorylation on Ser1177 [17]. Interestingly, the endothelial cell–specific overexpression of the insulin receptor in mice attenuated agonist‐induced endothelium‐dependent relaxation by initiating the Pyk2‐dependent tyrosine phosphorylation of eNOS on Tyr657. Importantly, Pyk2 inhibition improved both the insulin‐ and shear stress–induced activation of eNOS [54]. The Pky2‐ and tyrosine phosphorylation–dependent inhibition of eNOS has also been implicated in the decrease in NO bioavailability after angiotensin II administration [18], ischemia–reperfusion injury [55, 56] and the development of atherosclerosis [19]. The regulation of eNOS activity by increased Tyr657 phosphorylation in situations associated with vascular dysfunction is an attractive hypothesis especially as large clinical trials with antioxidant therapies have failed to show a beneficial effect on cardiovascular outcome [57]. Moreover, there are a large number of reports that are seemingly incompatible with a major role of BH4‐dependent eNOS uncoupling in vivo (for review, see [21]). Although NO output has been successfully improved by enhancing cellular levels of BH4, either using sepiapterin or by preventing its oxidation, the association of Hsp90 with eNOS [58], as well as eNOS phosphorylation, can also impact on the balance of NO/O_2_^−^ production. A second tyrosine residue (Tyr81, human sequence) has been linked to agonist‐induced eNOS activation, most probably by promoting protein–protein interactions [59]. Tyr81 was found to be phosphorylated by Src [59] and Abl [60] and dephosphorylated by vascular endothelial protein tyrosine phosphatase (VE‐PTP), which is of relevance in as much as VE‐PTP expression is increased by hypoxia [61] and in metabolic conditions such as diabetes [62]. Indeed, VE‐PTP inhibition increased eNOS activity to improve endothelial function and decrease blood pressure by increasing the phosphorylation of eNOS on Tyr81 as well as Ser1177 [60].

## 4 How NO Works

**FIGURE 1:**
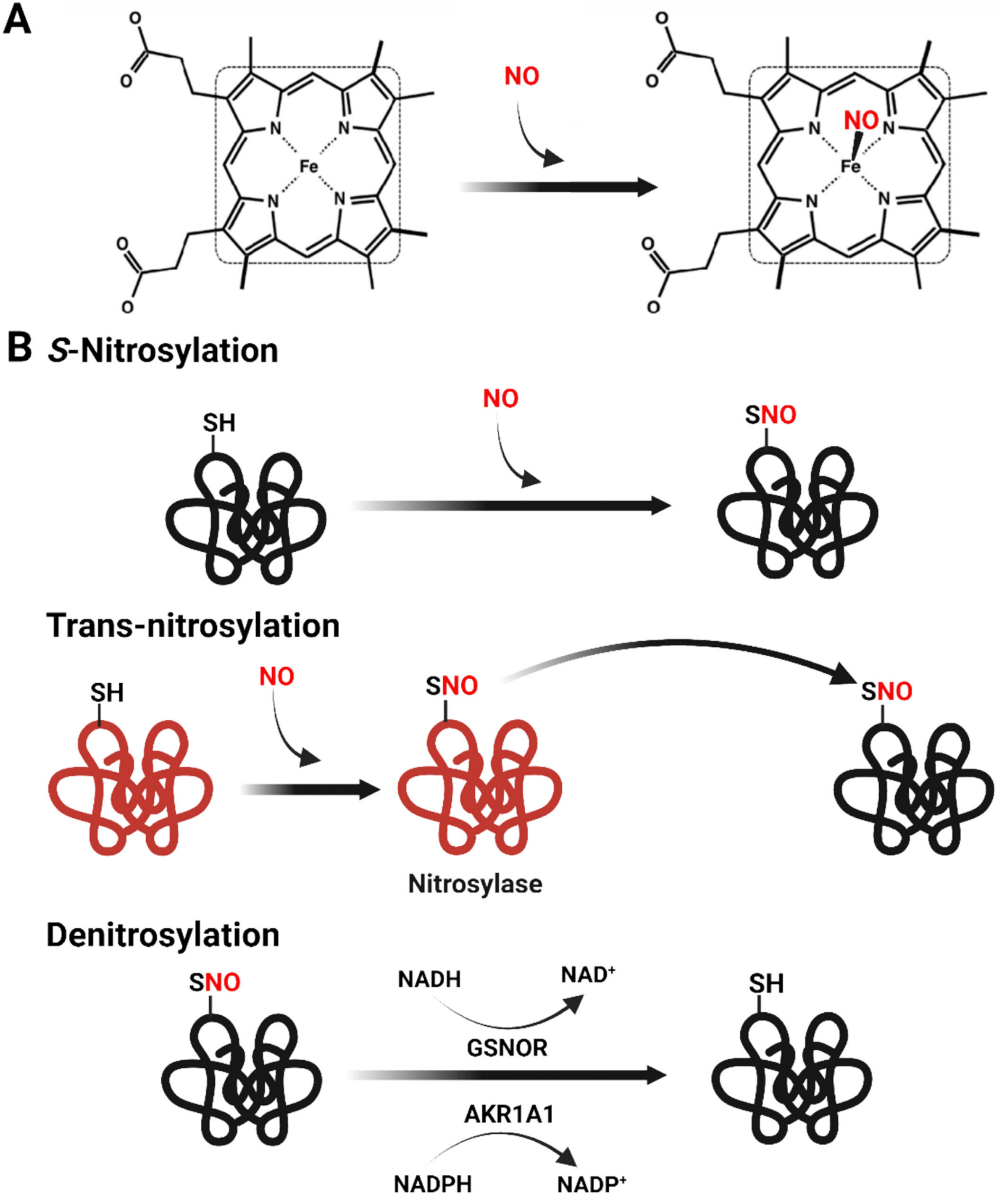
How NO works. NO elicits its biological actions by (A) binding to redox active metals such as the haem iron in sGC, cytochrome C, or deoxygenated myoglobin and haemoglobin to form a metal–nitrosyl complex, or by its transfer to a reduced thiol (B) in a process referred to as *S‐*nitrosylation. *S‐*nitrosylated proteins have been proposed to expand the sphere of influence of NO by transferring it to secondary acceptor proteins in a process referred to as transnitrosylation. Such proteins are referred to as protein nitrosylases. *S‐*nitrosylation was initially presumed to take place nonenzymatically but growing evidence now suggests that nitrosylation and denitrosylation are enzymatically regulated. To date, the *S*‐nitrosoglutathione reductase (GSNOR) and aldo‐keto reductase family 1 member A1 (AKR1A1) have been classed as denitrosylases and reduce *S*‐nitrosothiols in a NADH and NADPH‐dependent manner, respectively. Created in BioRender. Aiad, M. (2025) https://BioRender.com/s68u524.

NO elicits its biological actions by binding to redox active metals such as the haem iron in sGC, cytochrome C or deoxygenated myoglobin and haemoglobin to form a metal–nitrosyl complex [63, 64], or by its transfer to a reduced thiol in a process referred to as *S‐*nitrosation or *S‐*nitrosylation [65]. The ability of NO to inhibit NF‐κB to attenuate the induction of inflammatory genes was initially attributed to the induction, stabilization and nuclear translocation of its inhibitor, IκBα [66, 67]. However, it is now clear that the p50 and p65 subunits of NF‐κB can be *S‐*nitrosylated to prevent their binding to DNA [68, 69, 70]. It is now widely recognized that NO signalling via reversible protein *S‐*nitrosylation is a rapidly initiated important posttranslational modification that regulates the activity of an ever increasing number of proteins (for reviews, see [4, 5, 71]), including the β1 subunit of sGC [72] (Figure 1). *S‐*nitrosylation is a modification that is not restricted to cells expressing the high NO output iNOS isoform but can also be detected in agonist‐stimulated endothelial cells in situ [73].

*S‐*nitrosylation was initially presumed to be driven solely by the chemical reactivity of nitrosylation agents and thiol groups. Several mechanisms were suggested for *S‐*nitrosylation including (i) the transformation of NO into N_2_O_3_, which then reacts with protein thiols, (ii) the conversion of NO to its radical form (NO·), which binds to the protein thiyl radical, or (iii) NO oxidation into NO_2_, which then reacts with thiols (for review, see [71]). More recent work suggests that nitrosylation and denitrosylation are enzymatically regulated. Also, *S‐*nitrosylated proteins have been proposed to transfer NO to a second acceptor protein in a process referred to as protein–protein transnitrosylation [74]. Determinant for *S‐*nitrosylation is the local cysteine environment, that is, its accessibility as well as the presence of acidic and basic residues as demonstrated by the transfer of NO groups from low‐mass *S‐*nitrosothiols to Cys93 in the β‐subunit of haemoglobin [75]. The likelihood that a specific cysteine undergoes *S‐*nitrosylation is determined by the concentration of the NO donor around the target cysteine residue [76]. Location is a large determinant of effective *S‐*nitrosylation and although a proportion of S‐nitrosylated proteins do not associate with a NOS isoform [77], many modified proteins in endothelial cells actually interact with eNOS including caveolin 1 [78, 79] and dynamin [80], as well as Hsp90 [81] and its client Akt [82].

Proteins that can transfer their NO groups to secondary target proteins are referred to as ‘protein nitrosylases’, much like protein kinases that attach to target proteins to add phosphate groups [83, 84]. In general, metalloproteins may function as *S‐*nitrosylases [82, 85, 86]. In this manner, haemoglobin functions as an erythroid anion exchanger 1 nitrosylase [87], caspase 3 acts as a nitrosylase for X‐linked inhibitor of apoptosis (XIAP) [88], and thioredoxin‐1 may be a caspase‐3 nitrosylase [89]. In cells, one of the most concentrated NO acceptors is glutathione, which makes *S‐*nitrosoglutathione (GSNO), a frequent NO donor [90]. The first indication that *S‐*nitrosylation is an enzymatic reaction came from studies in anaerobically grown *Escherichia coli* that identified the hybrid cluster protein as being responsible for most cellular protein *S‐*nitrosylation and to prime cells to become resistant to subsequent nitrosative stress [91]. Importantly, hybrid cluster protein was discovered to be part of a protein complex together with nitrate reductase and transnitrosylases that promoted the propagation of NO signalling [92].

Denitrosylases couple NADPH/NADH oxidation with reduction of *S‐*nitrosothiols, with the GSNO reductase (GSNOR, *Adh5*) reported to account for most NADH‐dependent GSNOR activity [93, 94], and aldo‐keto reductase family 1 member A1 (AKR1A1), was identified as an NADPH‐dependent mammalian SNO‐CoA and GSNO reductase [95]. AKR1A1 was subsequently reported to associate with the endoplasmic reticulum secretory machinery to control an *S‐*nitrosylation cascade involving the cargo‐selection proteins SAR1 and SURF4. SAR1 was found to *S‐*nitrosylate SURF4, which in turn *S‐*nitrosylated PCSK9 to inhibit its secretion. AKR1A1 counteracted *S‐*nitrosylase activity by promoting PCSK9 denitrosylation [96]. Indeed, mice deficient in AKR1A1 were reported to exhibit marked reductions in serum cholesterol because of reduced secretion of the cholesterol‐regulating protein PCSK9 [96]. An inhibitor of AKR1A1 decreased SNO‐CoA metabolic activity in multiple organs and protected against acute kidney injury through the *S‐*nitrosylation of pyruvate kinase M2 (PKM2) [97].

Recent work suggests that *S‐*nitrosothiols are unstable intermediates that tend to form disulphides either with other cysteines in the protein or with free glutathione (GSH). Indeed, the *S*‐nitrosylation of several proteins is reported to alter disulphide bonds [98].

## 5 *S‐*Nitrosylated Proteins

Initially, the identification of *S‐*nitrosylated proteins relied on the biotin switch method that was restricted to a targeted approach. Despite this, a large number of *S‐*nitrosylated proteins were identified with functions in the regulation of cell adhesion, angiogenesis and metabolism. The more wide spread accessibility to mass spectrometry has accelerated the identification of *S‐*nitrosylated proteins [71, 99, 100, 101, 102], and these are now known to include Ca^2+^ channels; like the ryanodine receptor [103], L‐type Ca^2+^ channel α1 subunit [104] and canonical transient receptor potential channels [105], as well as cytoskeletal proteins [106], proteins involved in metabolism, apoptosis and autophagy in cancer [76, 107, 108], Golgi and nuclear proteins [109, 110, 111]. A prototypical *S‐*nitrosylated protein is haemoglobin, which in mammalian cells can function as both SNO synthase and nitrosylase [112], a topic that has been reviewed in detail elsewhere [113, 114].

Given that eNOS is concentrated in the Golgi apparatus, it is not surprising that some of the best characterized *S*‐nitrosylated proteins are Golgi or Golgi‐associated proteins [111]. One example of the latter is *N*‐ethylmaleimide–sensitive fusion protein (for review, see [115]), which is a member of the AAA family of ATPases that generally uses ATP hydrolysis to alter the conformation of a substrate protein. When this protein is *S*‐nitrosylated, its ability to interact with other proteins is reduced, and this results in impaired protein transport from the endoplasmic reticulum to the plasma membrane [116] as well as its ability to bind to SNARE proteins and regulate the exocytosis of Weibel–Palade bodies [117]. In platelets, this results in impaired platelet granule exocytosis [118]. Many nuclear proteins are also reported to be *S‐*nitrosylated. As eNOS was thought to be restricted to either the plasma membrane or the Golgi apparatus, this process was presumed to be indirect, which implied that transnitrosylation was the most likely mechanism by which nuclear proteins were modified. Indeed, the *S‐*nitrosylation of GAPDH facilitates its interaction with nuclear transport proteins like Siah1, to translocate to the nucleus. Once there, *S‐*nitrosylated GAPDH can transfer NO to nuclear proteins, such as histone deacetylases and sirtuins. This modification inhibits the activity of these nuclear proteins, affecting their roles in transcriptional regulation, chromatin remodelling and cellular stress responses [119]. However, eNOS is also present in the endothelial cell nucleus [109, 120], and 81 nuclear eNO*S‐*interacting proteins, including RNA‐binding proteins involved in RNA processing, paraspeckle formation and double‐stranded RNA (dsRNA) interactions were identified [109]. Importantly, about 80% of these interacting proteins were already listed in available *S‐*nitrosylation databases generated using other cell types. Given the large number of *S*‐nitrosylated proteins identified to date, it is clear that NO can influence an impressive number of different cellular functions. The following section will focus more on the link between NO and metabolism and cardio‐metabolic disease.

### 5.1 Angiogenesis

Classical actions of NO relate to its impact on cell proliferation and angiogenesis as well as VEGF‐induced changes in permeability. There have been numerous reports of essential angiogenic signalling molecules being directly modified by NO [4]. For example, hypoxia enhances protein *S‐*nitrosylation in endothelial cells and targets proteins such as Ras‐GTPase–activating protein and protein disulphide isomerase, among many others [121]. Also, VEGF stimulates NO generation and the *S‐*nitrosylation of the protein tyrosine phosphatase, SH‐PTP1, to decrease its activity and attenuate the dephosphorylation of its substrate VEGF receptor‐2 [122]. The increase in vascular permeability induced by VEGF has further been linked to the *S‐*nitrosylation of β‐catenin to prevent its dissociation from VE‐cadherin and thus the disassembly of adherens junction complexes [123], as well as its association with T‐cell factor 4 to inhibit endothelial cell proliferation by Wnt3a [124]. Even the activity of vasodilator‐stimulated phosphoprotein, which is dependent on cGMP/cAMP and G/A kinases, can be inhibited by its *S‐*nitrosylation to increase endothelial permeability [125]. Although not yet directly linked to angiogenesis, the finding that β‐arrestin can be *S*‐nitrosylated to account for altered β‐adrenergic responsiveness [126] can potentially impact on the internalization and signalling of other key endothelial cell receptors.

### 5.2 Metabolism

A clear link between NO and the regulation of cellular metabolism and cardio‐metabolic disease has been established. For example, modulation of the S1176 phosphorylation site resulted in unanticipated effects on insulin sensitivity, energy metabolism, and body weight regulation [51]. Also, preventing the phosphorylation of the Tyr657 site attenuated atherogenesis and helped to maintain endothelial cell levels of reducing equivalents to combat oxidative stress [19]. Specific metabolic pathways are targeted by NO. To date, several metabolic enzymes in different pathways were found to be modulated by S‐nitrosylation.

Several glycolytic enzymes can be *S‐*nitrosylated, including glyceraldehyde‐3‐phosphate dehydrogenase [127], phosphofructokinase [128] and PKM2 [19]. However, whether all of these can be targeted by the constitutive NOS isoforms is not entirely clear. The *S‐*nitrosylation of glycolytic proteins can impact on associated pathways. For example, the *S‐*nitrosylation of PKM2 was shown to inhibit the activity of the enzyme and to redirect glucose from glycolysis to the pentose phosphate pathway to replenish levels of the reducing equivalents NADPH and GSH [19]. A decrease in PKM2 *S‐*nitrosylation was associated with vascular dysfunction and decreased the levels of reducing equivalents. Importantly, in mice in which eNOS Tyr656 (murine sequence) was replaced with phenylalanine to prevent the PYK2‐dependent inactivation of the enzyme, GSH levels were maintained to combat oxidative stress and atherogenesis [19]. *S‐*nitrosylation of PKM2 has also been linked to protection against acute kidney injury [97]. Numerous mitochondrial proteins involved in energy and redox regulation, transport, iron homeostasis, translation, mitochondrial morphology and apoptosis are reported to be *S‐*nitrosylated [129]. The regulation of mitochondrial function by NO has been attributed to the mitochondrial translocation of eNOS and/or the presence of a NOS isoform in mitochondria (for reviews, see [115, 130]). Perhaps the best studied is cytochrome C oxidase (Complex IV) as it is a terminal enzyme of the mitochondrial electron transport chain and is essential enzyme for regulating energy production. NO *S*‐nitrosylates active site cysteines in complex IV, which results in its persistent inhibition [131]. NO also impacts on fatty acid synthesis as it can modify the acetyl‐CoA carboxylase, the rate‐limiting enzyme in de novo lipogenesis [132]. *S‐*nitrosylation increases acetyl‐CoA carboxylase activity and high fat diet–induced metabolic function was reported to decrease hepatic eNOS expression and acetyl‐CoA carboxylase *S‐*nitrosylation thereby likely counteracting hepatic lipid deposition [132]. In the brain, NO and *S‐*nitrosylation are reported to regulate glutamine/glutamate metabolism at least partly by targeting the astrocyte glutamate transporter GLT1 to reduce glutamate uptake [133]. It is unclear whether or not eNOS‐derived NO can elicit a similar effect in endothelial cells, which have a high dependence on glutamine metabolism [134].

## 6 Regulation of Gene Expression

NO regulates gene expression through multiple mechanisms.

### 6.1 Transcription Factors

A number of transcription factors can be *S‐*nitrosylated including the AP1 subunit c‐jun [135], the p50 and p65 subunits of NF‐κB [68, 69, 70] and human elongation factor‐1‐delta [121]. Similarly, the tumour suppressor protein p53 undergoes *S‐*nitrosylation and is required for the induction of antioxidant genes in gastrocnemius/soleus muscles, a process that was impaired during ageing [136]. Other examples include hypoxia‐inducible factors (HIFs) and oestrogen receptors (ERs). For example, in aggressive prostate cancer, the eNOS forms complexes with HIFs and ERβ to regulate hypoxia‐responsive elements on DNA. This complex interaction induces chromatin remodelling and the transcription of genes associated with tumour progression, illustrating role of NO in a hypoxic microenvironment [137]. The combinatorial action of eNOS and ERα on the human telomerase reverse transcriptase (hTERT) promoter demonstrates how NO interacts with oestrogen‐responsive elements to regulate telomerase activity. This regulation is vital for angiogenesis and cellular longevity, showcasing the role of NO in transcriptional specificity [138]. These examples highlight the ability of NO to regulate diverse transcription factors, contributing to its broad influence on gene expression and cellular responses.

### 6.2 Epigenetic Regulation

NO exerts significant control over the epigenetic landscape. It regulates histone modifications, DNA methylation and microRNA levels, thereby modulating gene expression. Histone acetyltransferases (HATs) play a crucial role in acetylating lysine residues on histone tails, a modification generally linked to gene activation. NO indirectly modulates HAT activity through interactions with proteins such as glyceraldehyde‐3‐phosphate dehydrogenase (GAPDH). When *S‐*nitrosylated, GAPDH localizes to the nucleus and associate with p300/CBP, a prominent HAT complex. This interaction enhances p300 auto‐acetylation and catalytic function, creating a feedback loop that sustains acetylation levels [139]. Studies in oral cancer cells exposed to NO donors like GSNO demonstrated increased acetylation of histones H3 and H4, correlating with higher p300 activity [140]. Deacetylases (HDACs), responsible for removing acetyl groups from histones, are also regulated by NO. *S‐*nitrosylation of HDAC2 and HDAC3 has been reported, although their catalytic activities remain unaffected [141, 142, 143]. Such modifications may influence their interactions with other chromatin‐associated proteins. In models of inflammation, inhibiting NO production reduced histone acetylation, highlighting the role of NO in maintaining the dynamic balance between acetylation and deacetylation [143].

NO also regulates histone methylation patterns by affecting lysine demethylases, which remove methyl groups from histone lysine residues. For instance, NO can inhibit Jumonji C domain–containing demethylases, enzymes dependent on iron and α‐ketoglutarate for their function. By disrupting the metal centres of these enzymes, NO alters histone methylation states [144]. This inhibition contributes to changes in gene expression profiles associated with inflammation. Added to all of this, NO can *S‐*nitrosylate the acyl‐coenzyme A (acyl‐CoA) species, which are cofactors for the acetyltransferases that posttranslationally modify thousands of proteins [145]. Also, a SNO‐CoA–assisted nitrosylase (SCAN) has been reported to use SNO‐CoA to *S‐*nitrosylate multiple proteins in the insulin signalling cascade including the insulin receptor and insulin receptor substrate 1. Insulin was reported to stimulate the *S‐*nitrosylation of both of the latter proteins to attenuate insulin signalling. The hypernitrosylation of the proteins, which was reported in obesity, promoted skeletal muscle insulin resistance and mice lacking SCAN were protected from diabetes [146].

### 6.3 RNA Editing

One of the proteins that physically associated with nuclear eNOS was the double‐stranded RNA‐specific adenosine deaminase (ADAR1). This is an enzyme that catalyses the conversion of adenosine (A) to inosine (I) in double‐stranded RNA (dsRNA) through a process called RNA editing [147]. This diversifies the transcriptome by altering codons, influencing splicing and modulating RNA stability and protein function. RNA editing also plays a critical role in regulating innate immunity, neuronal activity and responses to viral infections [148]. Probably the most important physiological role of ADAR1 is to edit endogenously generated dsRNAs, which are immunogenic, to keep amounts low under physiological conditions. Were this not to happen, the accumulation of dsRNAs would elicit a type I interferon (IFN) response [149, 150]. In line with this, endothelial cell–specific deletion of ADAR1 resulted in postnatal lethality because of the activation of innate immunity and multiorgan damage [151]. Altered ADAR1 activity in atherosclerosis has been linked to altered editing of the cysteine protease cathepsin S, to facilitate the recruitment of the stabilizing RNA‐binding protein human antigen R [152], as well as to altered expression of the long noncoding RNA *NEAT1* [153]. Similarly, inactivation of ADAR1 in cardiomyocytes resulted in late‐onset auto‐inflammatory myocarditis and heart failure triggered by the accumulation of unedited dsRNAs and activation of type I IFN signalling and apoptosis [154]. There are several reports of endothelial cell dysfunction being linked to type I IFN signalling [155]. However, in most cases, responses were attributed to the activation of the IFN receptor in endothelial cells [156], rather than to the role of the endothelium in the initiation of the response. The finding that eNOS‐derived NO can *S‐*nitrosylate ADAR1 [109], made a direct link between endothelial dysfunction and vascular inflammation. Indeed, the deletion of eNOS to abrogate NO generation had a marked impact on endothelial cell RNA editing at the same time as eliciting the accumulation of dsRNA, the induction of IFN‐α and β as well as a marked downregulation of cell cycle‐related genes [109]. As a result, growth factor‐stimulated cell proliferation was abrogated and tumour necrosis factor‐α or H_2_O_2_‐induced cell death were increased. Similarly, endothelial dysfunction in mice and in patients with atherosclerosis was accompanied by the accumulation of dsRNA and the activation of the type I IFN signalling pathway. Preserving NO bioavailability in vivo fully prevented these effects [109]. This novel mechanism linking nuclear eNOS‐generated NO to ADAR1 function highlights the essential role of eNOS in preventing dysregulated type I IFN responses and maintaining endothelial homeostasis. One of the IFN‐regulated proteins upregulated by both eNOS and ADAR1 knockdown in human endothelial cells IFN‐stimulated gene 15 (ISG15); which plays an essential role as host‐defence response to microbial infection, was identified as a possible mediator of hypertension‐associated vascular damage. This is worth mentioning because ISG15 was identified using a bioinformatics approach and its expression correlated with systolic and diastolic blood pressure and carotid intima‐media thickness and was increased in aortae from hypertensive mice as well as in aortic aneurysms [155]. Moreover, mice deficient in Isg15 were protected against angiotensin II‐induced hypertension and vascular dysfunction [155], and ISG15 was one of the IFN‐regulated proteins upregulated by both eNOS and ADAR1 knockdown in human endothelial cells [109]. Linking endothelial dysfunction with type I IFN signalling may also play a role in other diseases such as Alzheimer's disease as, at least in a mouse model of the disease, type I IFN signalling has been linked to brain endothelial barrier dysfunction characterized by the downregulation of the adherens junction protein VE‐cadherin [157].

## 7 Conclusion

The fact that endothelium‐derived NO plays an important role in the regulation of vascular function is well known. There is a vast amount of evidence linking altered NO generation and vascular function with cardiovascular disease, but it is only relatively recently that a clear link between a genetic predisposition to enhanced NO signalling could be associated with a reduced risk of vascular disease in a patient population [3]. Technological advances in the mass spectrometry–based detection of *S*‐nitrosylated proteins has helped identify the cellular processes that are regulated by NO, with some of them being unexpected e.g. the link between eNOS and metabolism or RNA editing. It may be an old target, but the sheer impact of eNOS on vascular health really justifies a revaluation of therapeutic options to maintain and protect eNOS activity.

## Conflicts of Interest

The authors declare no conflicts of interest.

## Data Availability

Data sharing is not applicable to this article as no new data were created or analysed in this study.
